# Interferon-gamma inducible protein 10 (IP10) induced cisplatin resistance of HCC after liver transplantation through ER stress signaling pathway

**DOI:** 10.18632/oncotarget.4832

**Published:** 2015-08-12

**Authors:** Wei Geng, Chung-Mau Lo, Kevin T.P. Ng, Chang-Chun Ling, Xiang Qi, Chang-Xian Li, Yuan Zhai, Xiao-Bing Liu, Yuen-Yuen Ma, Kwan Man

**Affiliations:** ^1^ Department of Surgery, The University of Hong Kong, Hong Kong, China; ^2^ Collaborative Innovation Center for Diagnosis and Treatment of Infectious Diseases, Hangzhou, China; ^3^ Department of Transplantation and Hepatic Surgery, Renji Hospital, School of Medicine, Shanghai Jiaotong University, Shanghai, China; ^4^ Department of Surgery, David Geffen School of Medicine, University of California at Los Angeles, Los Angeles, CA, USA

**Keywords:** IP10, HCC, liver transplantation, cisplatin resistance, graft injury

## Abstract

Tumor recurrence remains an obstacle after liver surgery, especially in living donor liver transplantation (LDLT) for patients with hepatocellular carcinoma (HCC). The acute-phase liver graft injury might potentially induce poor response to chemotherapy in recurrent HCC after liver transplantation. We here intended to explore the mechanism and to identify a therapeutic target to overcome such chemoresistance. The associations among graft injury, overexpression of IP10 and multidrug resistant genes were investigated in a rat liver transplantation model, and further validated in clinical cohort. The role of IP10 on HCC cell proliferation and tumor growth under chemotherapy was studied both *in vitro* and *in vivo*. The underlying mechanism was revealed by detecting the activation of endoplasmic reticulum (ER) stress signaling pathways. Moreover, the effect of IP10 neutralizing antibody sensitizing cisplatin treatment was further explored. In rat liver transplantation model, significant up-regulation of IP10 associated with multidrug resistant genes was found in small-for-size liver graft. Clinically, high expression of circulating IP10 was significant correlated with tumor recurrence in HCC patients underwent LDLT. Overexpression of IP10 promoted HCC cell proliferation and tumor growth under cisplatin treatment by activation of ATF6/Grp78 signaling. IP10 neutralizing antibody sensitized cisplatin treatment in nude mice. The overexpression of IP10, which induced by liver graft injury, may lead to cisplatin resistance via ATF6/Grp78 ER stress signaling pathway. IP10 neutralizing antibody could be a potential adjuvant therapy to sensitize cisplatin treatment.

## INTRODUCTION

Liver transplantation is a promising treatment for the patients with hepatocellular carcinoma (HCC), the 3 year survival rate of liver transplantation for HCC was up to 80% and the 5 year survival rate reached 75% [1–3]. However, the tumor recurrence is still the most serious threat to HCC patients after liver transplantation. Although many adjuvant therapies were applied to minimize the recurrent rate, the 5-year tumor recurrent rates ranged from 8% to 56% [4–6]. Chemotherapy, as an alternative treatment for HCC, had a remarkable response rate in primary liver cancer [7–11]. However, it had no benefit on recurrent HCC patients [12]. The reason for this difference was still unclear.

Living donor liver transplantation presents greater risks of liver graft injury due to the small size of donor liver [13]. From an analysis of 16 clinical cohorts, the tumor recurrent rate is significantly higher due to the graft injury in living donor liver transplantation (LDLT) group compared with deceased donor liver transplantation (DDLT) group [14]. In our recent studies, we have demonstrated the significance of acute phase graft injury on late phase tumor growth and invasiveness after liver transplantation in animal models [15]. The inflammatory response resulted from hepatic I/R injury not only provided a favorable environment for tumor growth, but also promoted tumor cell invasiveness [16]. Furthermore, chemokine (C-X-C motif) ligand 10 (IP10) was identified as a distinct gene signature of acute-phase graft injury and late-phase tumor recurrence after liver transplantation [17]. Post-transplant enhanced IP10 signaling in small-for-size liver grafts not only directly promoted tumor cell proliferation and invasiveness [17], but also mobilized circulating endothelial progenitor cells into liver graft and further promoted tumor angiogenesis during liver tumor recurrence after liver transplantation [18].

IP10, as a secretory protein, is secreted by many types, including leukocytes, activated neutrophils, eosinophils [19], monocytes, epithelial cells, endothelial cells, stromal cells and keratinocytes in response to IFN-γ [17]. IP10 played crucial roles in interferon responses including attraction of activated lymphocytes, monocytes, T cells and NK cells to inflammatory area [20]. Highly expression of IP10 was found in infectious diseases, inflammatory [21, 22], autoimmune diseases [23], and variety of cancer diseases. IP10 and its downstream signals were considered to be potential therapeutic targets in attenuation of acute phase graft injury and prevention of tumor recurrence after liver transplantation using small-for-size graft [17]. Increasing evidence suggested that ER stress was associated with graft injury and HCC recurrence after transplantation, but no studies specifically focused on the underlying mechanism [24]. It is worthwhile to explore the crosstalk between graft injury induced IP10 overexpression and the activation of ER stress after liver transplantation.

The endoplasmic reticulum (ER) is a specialized organelle that plays a central role in biosynthesis, correcting protein folding, and posttranslational modifications of secretory and membrane proteins. The activation of ER stress not only played important roles in graft injury during organ transplantation [24, 25], but also regulated cisplatin-induced cell death and drug resistance [26]. There are contradictory reports regarding the role of ER stress response in cancer [27]. Although there were indications that ER stress may provide protection against cancer [28, 29], plenty of examples suggested that the activation of ER stress signaling was essential for cancer cell survival and tumor recurrence [30, 31]. Tumor recurrence requires the residue circulating HCC cells which escaped from immune surveillance [32]. Recurrent HCC could also developed resistance to adjuvant therapies after liver transplantation [32]. However, the role of graft injury induced IP10 overexpression on post-transplanted chemoresistance is still unknown. As a distinct gene signature of acute-phase graft injury and tumor recurrence [17], IP10 might be the key factor bridging graft injury to chemoresistance in recurrent HCC after liver transplantation. IP10 neutralizing antibody might provide a potential adjuvant therapy to attenuate chemo-resistance in recurrent HCC.

In this study, we proposed that IP10 may promote cisplatin resistance after liver transplantation by activating ER stress signaling pathways. IP10 neutralizing antibody might sensitize cisplatin treatment in HCC.

## RESULTS

### Over expression of IP10 correlated with chemoresistance genes in rat liver transplantation model

**Figure 1:**
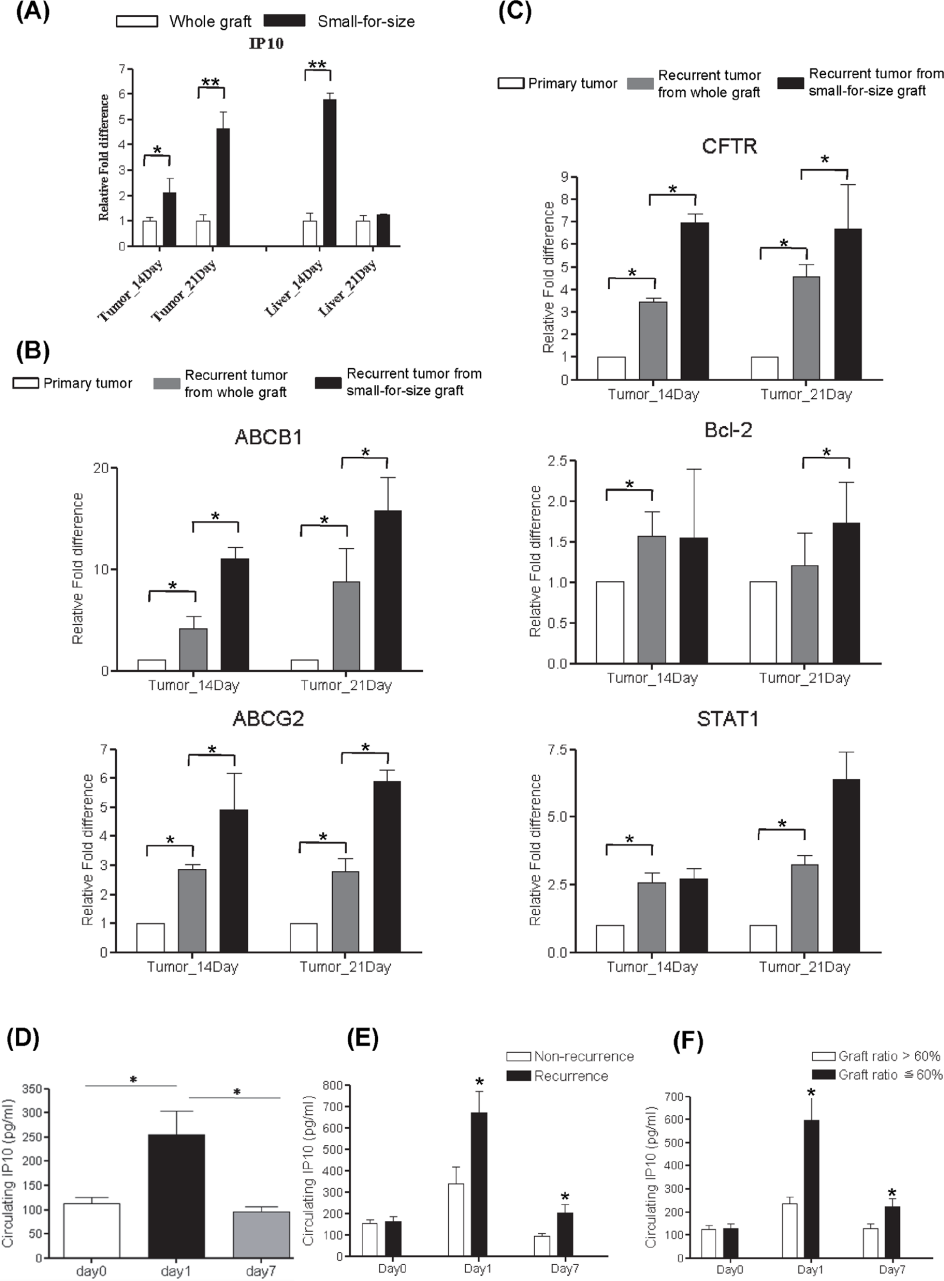
Over expression of Multidrug resistant genes in rat liver transplantation model and the expressions of IP10 in human liver transplantation **A.** mRNA level of ABCB1 and ABCG2 in tumor and liver samples from rat liver transplantation model. *P < 0.05. **B.** mRNA level of AKT and BCL2 in tumor and liver samples from rat liver transplantation model. *P < 0.05. **C.** mRNA level of STAT1 and CFTR in tumor and liver samples from rat liver transplantation model. *P < 0.05. **D.** Representative images of up-regulation of circulating IP10 in HCC patients at 1 day after liver transplantation. *P < 0.05. **E.** The association between tumor recurrence and IP10 expression level in clinical liver graft biopsies. *P < 0.05. **F.** The association between small graft ratio and intragraft IP10 expression level in clinical liver transplantation patients. *P < 0.05.

In rat liver transplantation model, IP10 was found to be overexpressed in tumor and liver tissues from small-for-size group at day 14 and day21 (Figure 1A). Several multidrug resistant genes including ABCB1, ABCG2, Bcl-2, CFTR and STAT1 were also overexpressed in small-for-size group compared with whole graft group (Figure 1B–1C).

#### Higher circulating IP10 correlated with tumor recurrence in HCC recipients

Significant elevation of circulating IP10 expressions was observed at acute phase (day 1) after liver transplantation by comparing with that at Day 0 and Day 7 (Figure 1D).

Circulating IP10 in recurrent group was significantly higher than that in non-recurrence group at day1 (677 ± 101 pg/ml *vs* 341 ± 93 pg/ml, *p* = 0.027) and day 7 (204 ± 32 pg/ml *vs* 123 ± 11 pg/ml, *p* = 0.039) (Figure 1E). Moreover, circulating IP10 expression in small-for-size group was significantly higher than that in whole graft group at day 1 (251 ± 53 pg/ml *vs* 119 ± 10 pg/ml, *p* = 0.013) and day 7 (251 ± 53 pg/ml *vs* 94 ± 11 pg/ml, *p* = 0.02) (Figure 1F).

#### IP10 induced cisplatin resistance in HCC cells

According to the expression level of IP10, six HCC cell lines were assigned into 2 groups, (1) lower IP10 expressed group (LO2, PLC HepG2 and MHCC97L) and (2) higher IP10 expressed group (Hep3B and Huh7) (Supplementary Figure S3).

#### Extracellular function of IP10 on HCC cell lines

IP10 recombinant protein (r-IP10) was applied to elevate the extracellular concentration of IP10 in cell culture environment. Elevation of extracellular IP10 significantly promoted HCC cell proliferation (Supplementary Figure S2).

**Figure 2:**
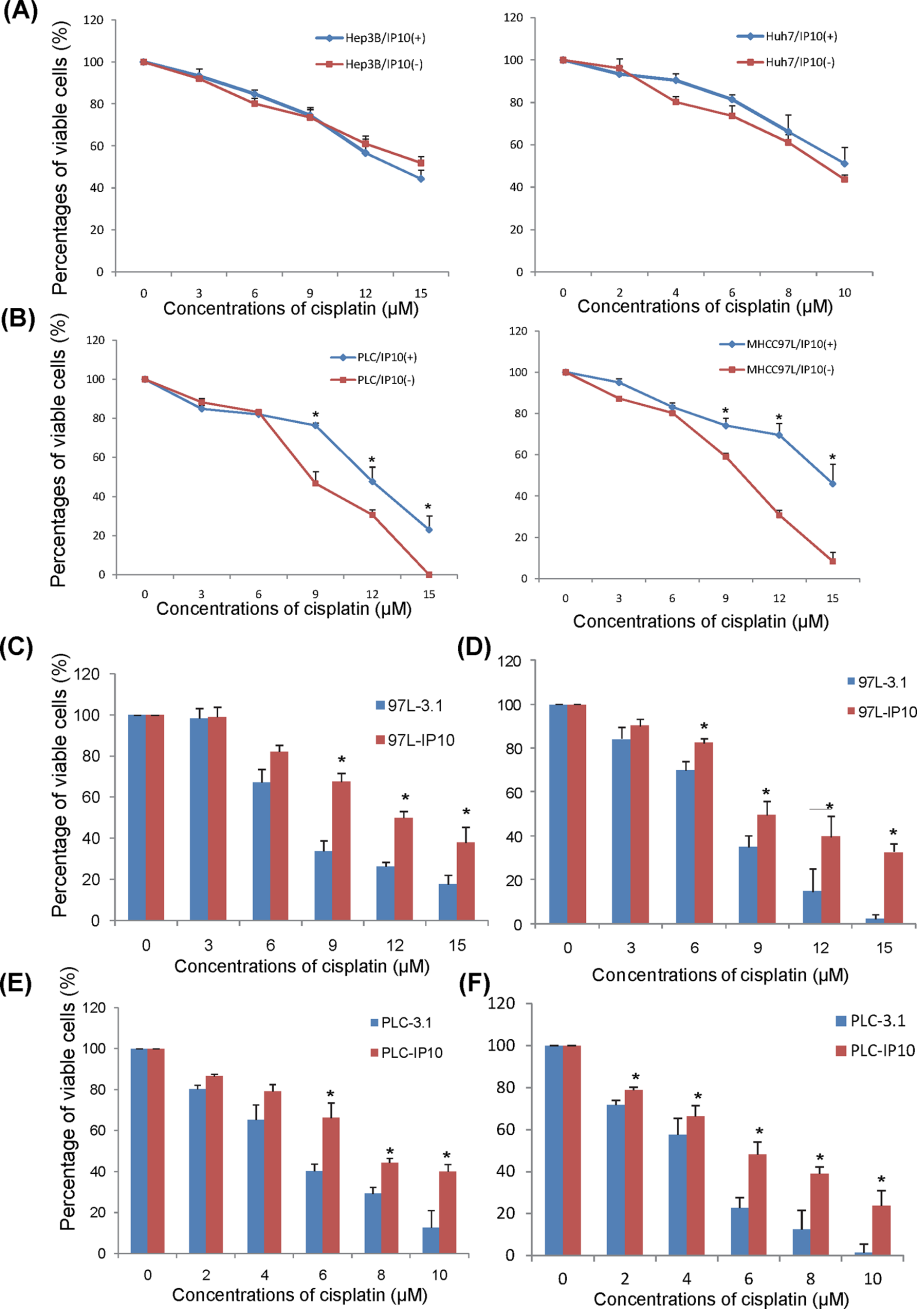
The effect of IP10 on HCC cell lines *in vitro* **A.** The effect of rIP10 administration on proliferation of Hep3B and Huh7 72 hrs by MTT assay. **B.** The effect of rIP10 administration on proliferation of PLC and MHCC97L under increasing concentrations of cisplatin for 72 hrs. *P < 0.05. **C.** The effect of cisplatin on proliferation of MHCC97L-3.1 and MHCC97L-IP10 was detected by MTT assay, *P < 0.05. **D.** The effect of cisplatin on proliferation of MHCC97L-3.1 and MHCC97L-IP10 was detected by colony formation assay. *P < 0.05. **E.** The effect of cisplatin on proliferation of PLC-3.1 and PLC-IP10 was detected by MTT assay, *P < 0.05. **F.** The effect of cisplatin on proliferation of PLC-3.1 and PLC-IP10 was detected by colony formation assay. *P < 0.05.

After 2 weeks of cisplatin administrated with/without r-IP10, there was no significant difference of cell proliferation rate in HCC cell lines with high expression of IP10—Hep3B and Huh7 (Figure 2A). r-IP10 could significantly promote HCC cell survive in PLC and MHCC97L under different concentrations of cisplatin treatment (Figure 2B).

#### Intracellular function of IP10 on HCC cell lines

The full length of IP10 was transfected into 2 HCC cell lines (PLC and MHCC97L) with low IP10 expression. The mRNA expression level of IP10 in stable transfectants were significantly higher than primary HCC cell lines, from 10 to 600 folds. Among them, PLC-IP10-3 and MHCC97L-IP10-1, with high IP10 expression were chosen for further studies (Supplementary Figure S4).

Several multi-drug resistant genes including ABCB1, ABCG2 and CFTR were up-regulated in both PLC-IP10 and MHCC97L-IP10. Expressions of MAP7, STAT1 and AKT also showed higher expression in IP10 stable transfectants (Supplementary Figure S5).

HCC cell proliferation rate was significantly higher in IP10 overexpression stable transfectant after 72 hours of cisplatin administration. When the concentration of cisplatin raised up to 9 μM, 12 μM and 15 μM, the percentages of viable cell of MHCC97L-IP10 was significantly higher compared to MHCC97L-3.1 (9 μM : 65.5 ± 5.7% *vs* 32.9 ± 6.6%, *p* = 0.027; 12 μM: 50.1 ± 4.3% *vs* 24.5 ± 1.9%, *p* = 0.019; 15 μM: 38.3 ± 9.1 *vs* 17.3 ± 6.4%, *p* = 0.035). The IC50 of cisplatin in MHCC97L-IP10 was around 1.6-fold higher than MHCC97L (Figure 2C). This result was also confirmed by colony formation assay (Figure 2D).

The percentages of viable cell of PLC-IP10 was significantly higher than PLC-3.1 under cisplatin administration (6 μM: 67.9 ± 10.1% *vs* 38.2 ± 4.3%, *p* = 0.04; 8 μM: 42.4 ± 2.7% *vs* 30.1 ± 4.0%, *p* = 0.035; 10 μM: 39.1 ± 4.7% *vs* 13.2 ± 11.5%, *p* = 0.031). The IC50 of cisplatin in PLC-IP10 was around 1.5-fold than PLC-3.1 (Figure 2E). This result was also confirmed by colony formation assay (Figure 2F).

In summary, overexpression of IP10 significantly promoted HCC cell proliferation and colony forming ability in PLC and MHCC97L HCC cell lines.

### IP10 promoted tumor growth under cisplatin treatment in animal models

#### In subcutaneous nude mice model

**Figure 3:**
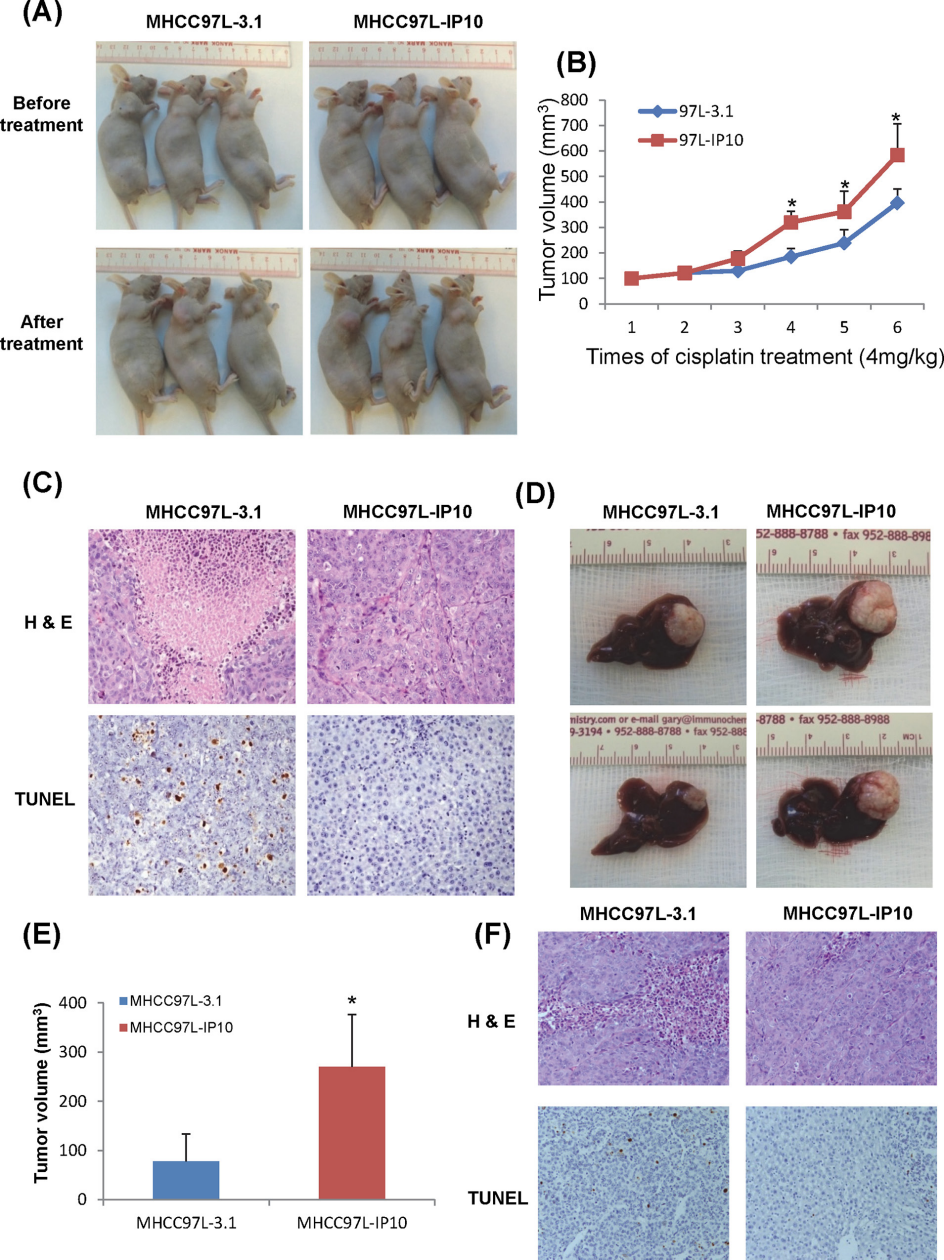
The effect of IP10 on tumor growth and chemoresistance in Subcutaneous and Orthotopic nude mice models **A.** The effect of IP10 stable transfectant (MHCC97L-IP10) on tumor formation under cisplatin treatment. The tumor volumes of each subcutaneous nude mouse model were recorded at the endpoint of this study. **B.** Tumor volumes were recorded each time after the cisplatin treatment. Representative images of tumor growth rate after cisplatin treatment. *P < 0.05 MHCC97L-IP10 *vs* MHCC97L-3.1 group. **C.** Representative images of tumor necrosis and tumor cell apoptosis by H & E and TUNEL staining in subcutaneous nude mice model (200×). **D.** Tumor volume at the endpoint of orthotopic nude mice model after cisplatin treatment. **E.** Representative images of significant higher tumor forming ability in MHCC97L-IP10 group under cisplatin treatment. *P < 0.05 MHCC97L-IP10 *vs* MHCC97L-3.1 group. **F.** Representative images of tumor necrosis and tumor cell apoptosis by H&E and TUNEL staining in orthotopic nude mice model (200×).

Average tumor volume from MHCC97L-IP10 was significantly larger than the control group after 3 weeks of cisplatin treatment (Figure 3A). Tumor growth rate was significantly higher in MHCC97L-IP10 group (Figure 3B). H&E and TUNEL staining demonstrated that tumor necrosis and tumor cell apoptosis was attenuated in MHCC97L-IP10 group (Figure 3C). These results demonstrated that IP10 overexpression could stimulate tumor growth and alleviate tumor necrosis, tumor cell apoptosis under cisplatin treatment.

#### In orthotopic liver tumor nude mice model

The tumor volume of MHCC97L-IP10 (268.3 ± 109.3 mm^3^) was significantly larger than the control group (90.2 ± 60.5 mm^3^) at the end point of this study (*p* = 0.041) (Figure 3D–3E). Tumor necrosis and tumor cell apoptosis was attenuated in MHCC97L-IP10 group (Figure 3F).

#### In orthotopic liver tumor nude mice model with hepatic IR injury

**Figure 4:**
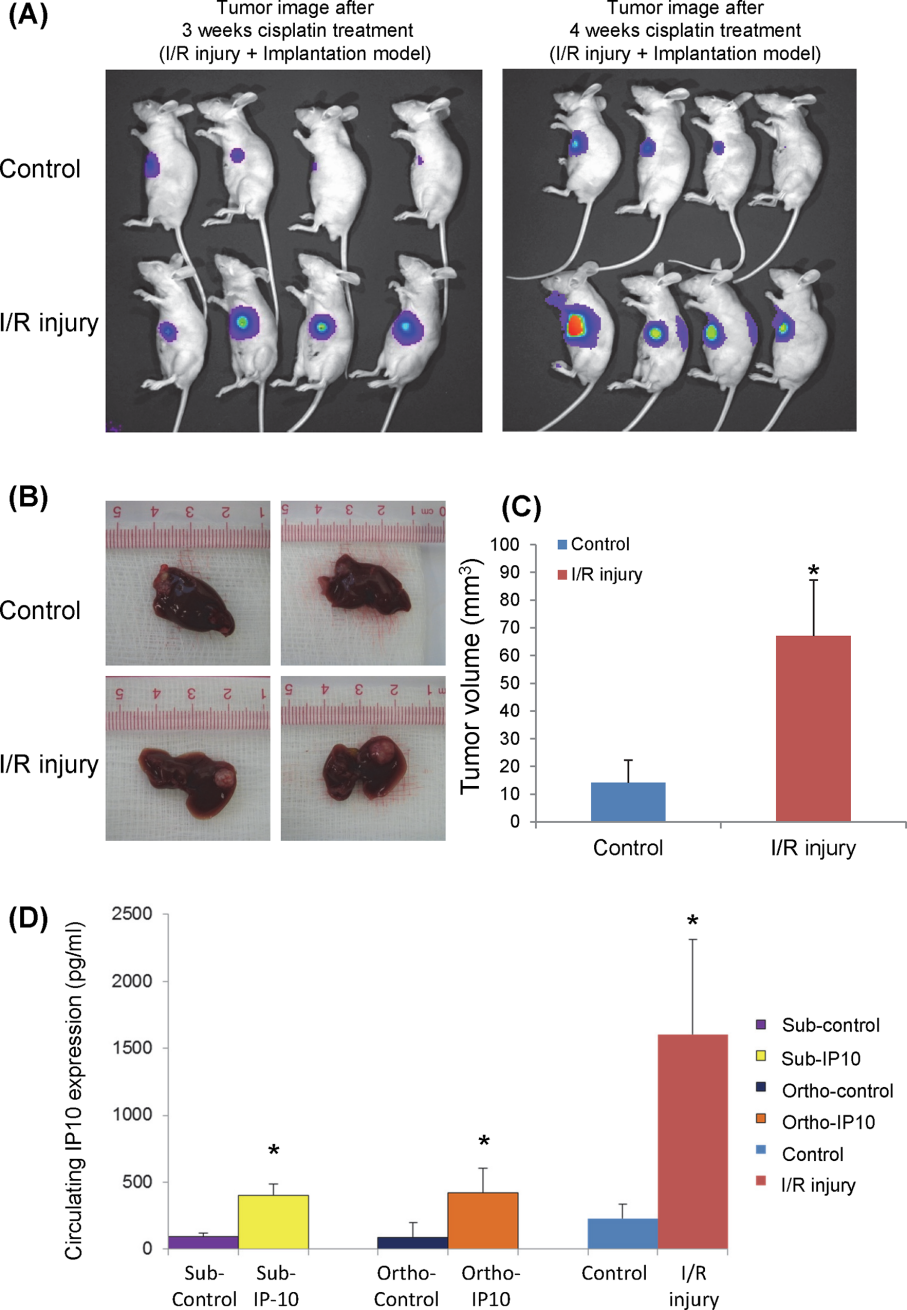
The effect of graft injury on IP10 expression of chemoresistance **A.** Optical image of tumor growth at 3 and 4 weeks cisplatin treatment. **B.** Tumor volume at the endpoint of orthotopic IR injury nude mice model after cisplatin treatment. **C.** Representative images of significant higher tumor forming ability in IR injury group under cisplatin treatment. *P < 0.05 vs control group. **D.** Comparison of circulating IP10 expression level in Subcutaneous, Orthotopic and IR injury nude mice models.

One group of nude mice was subjected to half an hour ischemia before tumor implantation. Cisplatin was given to these nude mice 2 weeks after tumor nodule implantation. According to the optical imaging, tumor size from IR injury group was larger compared to the control group after 3 and 4 weeks of cisplatin treatment (Figure 4A). The tumor volume was confirmed to be significantly larger in IR injury group by comparing with control group (14.9 ± 8.9 mm^3^ *vs* 65.5 ± 20.1 mm^3^, *p* = 0.01) (Figure 4B). The circulating IP10 expression in IR injury group was around 1700 pg/ml which was 9-fold of its expression in control group (Figure 4C). The circulating IP10 in IR injury nude mice models was significantly higher than subcutaneous and Orthotopic models. (Subcutaneous-IP10 group: 413.9 pg/ml; Orthtopic-IP10: 433.2 pg/ml; I/R group: 1672.3 pg/ml, *p* < 0.01) (Figure 4D)

In summary, IP10 overexpression was induced by hepatic IR injury. IP10 overexpression significantly promoted tumor growth and attenuated tumor cell apoptosis under cisplatin treatment.

### IP10 activated ER stress signaling pathway

ER stress includes three main pathways. One of them functions as anti-apoptotic effect with the key molecules ATF6/Grp78, other two pathways promote cell apoptosis by the activation of PERK-CHOP and IRE1α. In order to reveal the correlations between IP10 up-regulation and the activation of ER stress, the expressions of these key molecules were investigated in *in vitro*, *in vivo* and clinical samples.

#### In *in vitro* experiments

**Figure 5:**
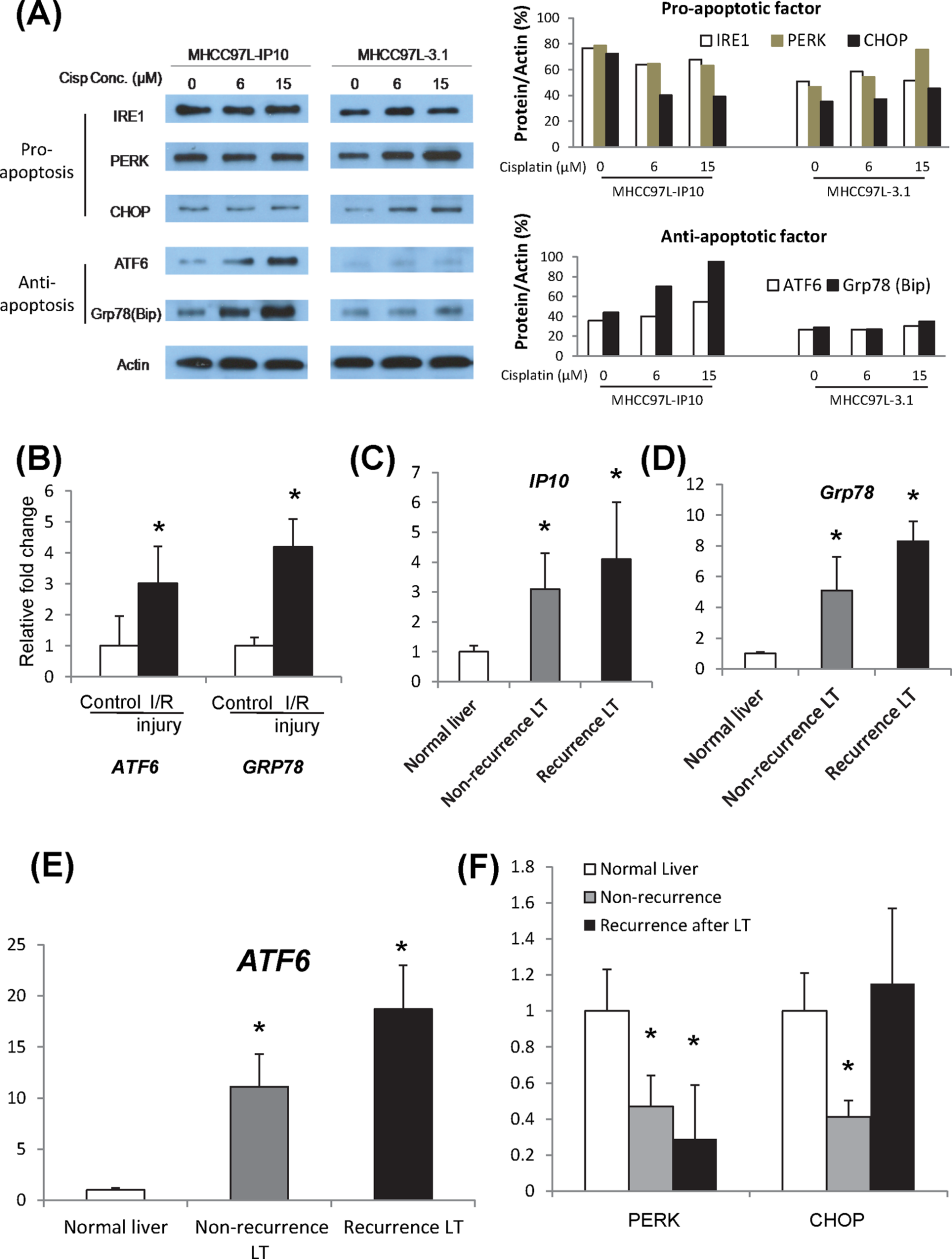
Activation of ER stress signaling pathways **A.** Activations of ER stress pathways, including CHOP, ATF6 and GRP78 in IP10 overexpressed stable transfectant (MHCC97L-IP10) after cisplatin administration. Right panel: Western-blot. Left panel: quantification analysis. **B.** mRNA level of GRP78 in tumor tissue from I/R injury nude mice model. *P < 0.05 vs control group. **C.** mRNA level of ATF6 in tumor tissue from I/R injury nude mice model. *P < 0.05 vs control group. **D.** mRNA level of PERK in tumor tissue from I/R injury nude mice model. **E.** mRNA level of CHOP in tumor tissue from I/R injury nude mice model. **F.** mRNA level of IRE1alpha in tumor tissue from I/R injury nude mice model.

MHCC97L-IP10 and MHCC97L-3.1 were administrated by different concentrations of cisplatin for 2 weeks. The expression of IP10, ATF6, Grp78, PERK, CHOP and IRE1 were examined. The anti-apoptotic factors (ATF6/Grp78) were significantly up-regulated by Cisplatin treatment in MHCC97L-IP10 group. It implied that over-expression of IP10 may induce chemo-resistance in HCC cells. Expressions of pro-apoptotic factors (PERK/CHOP) had a cisplatin dose dependant increasing in MHCC97L-3.1 group (Figure 5A). The expression of IRE1α did not have any significant difference.

#### In animal model

In nude mice liver tumor model with hepatic IR injury, Grp78 and ATF were found to be significantly overexpressed in tumor tissue. A positive correlation was observed between overexpression of IP10 and the activation of Grp78 and ATF6 (Figure 5B). However, expressions of PERK, CHOP, IRE1α and Caspase12 did not show any significant change (Supplementary Figure S5).

#### In clinical cohort

Intragraft IP10 was found to be overexpressed in HCC recipients, especially in the recurrent group (Figure 5C). Consistent with the up-regulation of IP10, the expression of Grp78 and ATF6 were significantly increased in liver tissues from the patients with HCC recurrence (Figure 5D–5E). The expression of PERK and CHOP was also up-regulated in the recurrent group (Figure 5F). There was no significant difference in the expression of IRE1α and Caspase12 (Supplementary Figure S5).

### IP10 neutralization antibody sensitized cisplatin treatment

#### In *in vitro* studies

**Figure 6:**
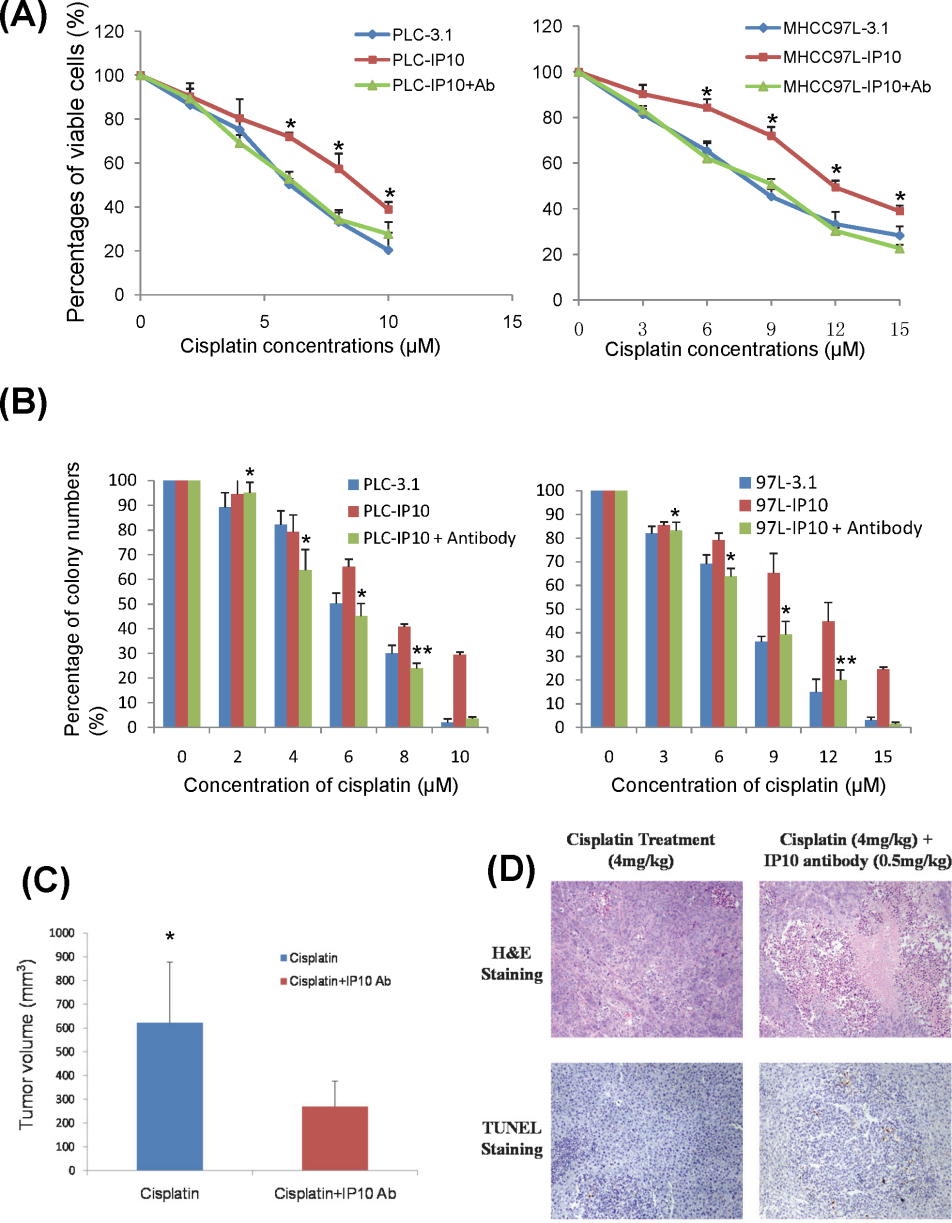
IP10 neutralization **A.** The effect of IP10 neutralizing antibody combined with cisplatin on proliferation of PLC and MHCC97L after 72 hrs was detected by MTT assay. *P < 0.05 vs cisplatin single treatment group. **B.** The effect of IP10 neutralizing antibody and cisplatin combined treatment on proliferation of PLC and MHCC97L by colony formation assay. *P < 0.05 vs cisplatin single treatment group. **C.** The comparison of tumor volume at the endpoint of orthotopic nude mice model after cisplatin combined with IP10 neutralizing antibody treatment. *P < 0.05 vs cisplatin single treatment group. **D.** Representative images of tumor necrosis and tumor cell apoptosis by H & E and TUNEL staining in orthotopic nude mice model with cisplatin and IP10 neutralizing antibody treatment (200×).

IP10 neutralizing antibody was applied as a combination treatment in PLC-IP10 and MHCC97L-IP10. HCC cell proliferation was significantly suppressed under cisplatin administration combined with IP10 antibody. The IC_50_ was significantly decreased in cells treated with the cisplatin and IP10 antibody (Figure 6A). IP10 antibody could also significantly suppress colony forming ability both in MHCC97L-IP10 and PLC-IP10 (Figure 6B).

#### In orthotopic nude mice liver tumor model

Fourteen days after tumor nodule implantation (Supplementary Figure S6), single cisplatin or combination with IP10 neutralizing antibody was given respectively. By comparing luciferin signal, the tumor growth was suppressed in IP10 neutralizing antibody combined with cisplatin group after 3 to 4 weeks of treatment. The tumor growth was significantly suppressed in combine treatment group by comparing with the cisplatin single treatment group (*p* = 0.012) (Figure 6C and Supplementary Figure S7). The treatment of cisplatin combined with IP10 neutralizing antibody significantly suppressed liver tumor growth by promoting tumor necrosis and apoptosis (Figure 6D). No obvious side effects related to IP10-antibody were observed during the observation period.

## DISCUSSION

IP10 plays a significant role in acute phase graft injury and has potential value to predict tumor recurrence after liver transplantation for liver cancer patients [17]. Overexpression of IP10 also contributed to HCC recurrence after liver transplantation through promoting of tumor cell proliferation and invasion, together with inducing angiogenesis by mobilization of circulating endothelial progenitor cells [17, 18]. In the current study, the acute phase up-regulation of IP10 was found to have significant correlations with graft injury and late phase tumor recurrent in HCC patients underwent liver transplantation. We firstly demonstrated that graft injury induced IP10 over-expression could promote cisplatin resistance in HCC cells by activation of ATF6/Grp78 ER stress signaling pathway. IP10 overexpression could activate ATF6/Grp78 both in *in vitro* and *in vivo* experiments, and further validated in our clinical cohort.

We also illustrated that over-expression of IP10 in HCC cells significantly promoted HCC cell proliferation and colony forming ability under cisplatin administration *in vitro*. In animal studies, three animal models were established to elaborate the relationships among IP10, graft injury and cisplatin resistance. IP10 could promote tumor growth and alleviate tumor necrosis under cisplatin treatment in both subcutaneous and orthotopic nude mice models. By comparing these three animal models, the IP10 up-regulation was induced by three different ways. Two of them were established by using IP10 stable transfectants and one was induced by IR injury. The result showed that the circulating IP10 expression was significant higher in IR injury model. In clinical samples, graft injury could induce the up-regulation of circulating IP10, which had significant correlations with HCC recurrent rate and small graft ratio. These data indicated that IP10 may have prognostic value to predict tumor progression and recurrence. It also confirmed the significant effect of IR injury on IP10 overexpression which could further promote tumor growth and invasiveness under cisplatin treatment. Cisplatin-induced DNA damage activates various signaling pathways to prevent or promote cell death, predominantly via apoptosis [33]. It was recently demonstrated that cisplatin could induce endoplasmic reticulum stress (ER stress) and non-nucleus-dependent apoptotic signal activation [34]. Currently, ER stress was considered to play crucial roles in cisplatin-induced tumor cell death as a cell stress signaling receptor. By examining the correlations between IP10 and the activation of ER stress, we may understand the underlying mechanism of IP10 overexpression induced chemo-resistance in HCC.

ER stress was regulated by the unfolded protein response (UPR) system. When the unexpected unfolded proteins were limited in the tolerable range, the UPR system could activate to restore the balance. If the stress was continuous, the UPR system could not restore the balance in prolonged ER stress and conduct programming death itself [35]. However, cancer cells could adapt to chronic stress in the tumor microenvironment by inducing the expression of GRP78/BiP, a major endoplasmic reticulum chaperone and anti-apoptotic properties. These residue cancer cells were responsible for tumor recurrence and associated with an unfavorable prognosis. In this study, IP10 was identified to play a key role in mediating the activation of ER stress and assisting post-transplant HCC cell survival via ATF6/Grp78 in our *in vitro* and *in vivo* experiments, further validated clinical samples. Graft injury induced IP10 overexpression was firstly demonstrated to be significantly correlated with the activation of ATF6/Grp78, which were key molecules in the anti-apoptotic pathway of ER stress signaling. IP10 might urge HCC cell survive from the ER stress and these cells might become more aggressive and resistant to chemotherapy due to the activation of ATF6/Grp78. However, several other proteins were also found to be up-regulated by graft injury, such as GRP19, IL-6 and HSP70 [17]. It will be worthwhile to investigate the role of those graft injury related genes on the late phase consequence after liver transplantation.

To date, more and more neoadjuvant therapies including the neutralizing antibodies, were applied as single or combined treatment for variety of cancer diseases. As an example, VEGF antibody (Bevacizumab) was found to have anti-tumor effect on colorectal cancer and HCC [36, 37]. Moreover, Bevacizumab was demonstrated to be an efficient adjuvant therapy to sensitize chemotherapy and achieve better outcome in advanced colorectal cancer and HCC [37, 38]. In this study, we firstly demonstrated that IP10 neutralizing antibody could be an efficient combination treatment to sensitize cisplatin treatment both *in vitro* and *in vivo*. It suggested the therapeutic potential of IP10 antibody treatment for HCC patients. However, the effect of IP10 neutralizing antibody was only investigated on cisplatin in current study. It will be worthwhile to further explore the application of IP10 antibody on other chemodrugs including 5-FU, doxorubicin and others. Additionally, by thorough understanding of the interaction between IP10 and ER stress activation, the downstream targets such as ATF6 and Grp78 will also have a potential value to be further studied whether they could sensitize chemotherapy and achieve a better outcome.

In conclusion, acute phase liver graft injury could induce the overexpression of IP10 which responsible for cisplatin resistance via ATF6/Grp78 ER stress signaling pathway. IP10 neutralizing antibody could be a potential adjuvant therapy to sensitize cisplatin treatment. It will provide a new angle for attenuating early-phase graft injury and the treatment of recurrent HCC patients.

## MATERIALS AND METHODS

### Clinical association study

#### Clinical specimens

The study was approved by the Institutional Review Board of the University of Hong Kong / Hospital Authority Hong Kong West Cluster.

Fifty HCC patients (30 patients within and 20 patients beyond Milan criteria; 36 patients within and 14 patients beyond UCSF criteria) who have undergone liver transplantation between May 2001 and April 2009 were recruited with informed consent from Department of Surgery, Queen Marry Hospital, The University of Hong Kong. Among them, 20 HCC patients had tumor recurrence after liver transplantation. Plasma samples were collected one day before the liver transplantation and 1 day, 7 days after liver transplantation. All these plasmid were stored at −80°C. Forty-eight liver samples were collected at 2 hours after portal vein reperfusion during surgery. Four donor samples were used as normal liver control. Patients with tumor recurrence were classified as tumor recurrent group. Other patients without tumor recurrence were assigned to non-recurrent group.

### Animal models

#### Animals

Male inbred Buffalo rat weights from 280 ~ 350 g were applied as donors and recipients for rat liver transplantation model. Male nude mice around 4~8 weeks old and weights from 20 ~ 25 g were applied for orthotopic and subcutaneous xenograft nude mice models. These buffalo rat and nude mice were housed in a standard laboratory environment with sufficient water, chow and free activity. They were kept under constant environment with a 12-hour light/dark cycle. They were fasted 12 hours before operation. All the operations were performed under sterilized condition. All the animal studies were approved by the Committee on the Use of Live Animals in Teaching and Research (CULATR), The University of Hong Kong.

#### Rat liver transplantation model

The procedure for the donor operation was described in our previous study [18].

#### Xenograft ectopic nude mice liver cancer model

Xenograft subcutaneous nude mice model was established to study the role of IP10 on induction of cisplatin resistance. Method was described in previous study [39]. All the mice were segregated randomly and six mice were recruited in each group.

#### Xenograft orthotopic nude mice liver cancer model

In order to study the effect of IP10 neutralizing antibody, an orthotopic nude mice model was established to compare the effect of cisplatin treatment alone and IP10 neutralizing antibody combined treatment (Supplementary Figure S1). All the mice were segregated randomly and six mice were recruited in each group.

Orthotopic nude mice liver cancer model with MHCC97L-3.1, MHCC97L-IP10 or MHCC97L-luc cell was established to observe the tumor growth under regular cisplatin treatment. Briefly, approximately 1 × 10^7^ cells in 0.2 ml of a culture medium were injected subcutaneously into the right flank of Balb/c nude mice. The mice were observed daily for signs of tumor development. Once the subcutaneous tumor had reached 1 cm in diameter, it was removed and cut into 1–2-mm cubes, which were then implanted into the left lobe of another group of nude mice (4 weeks old) [25]. Cisplatin or cisplatin with IP10 antibody treatment started at 2 weeks after the implantation. All the nude mice were segregated randomly. Four milligrams per kilogram cisplatin with/without 0.5 milligrams per kilogram IP10 antibody was injected intraperitoneally into the nude mice every 4 days and lasted for 4 weeks. Finally, the mice were anesthetized by intraperitoneal injection of pentobarbital (Abbott Laboratories Chicago, IL, USA) at a dose of 50 mg/kg. The volume of the tumors was measured and calculated (Volume = 1/2 × Length × Width^2^). The liver and tumor samples were collected and preserved in formalin and liquid nitrogen.

In order to simulate graft injury induced IP10 overexpression and its effect on developing chemoresistance in HCC, an orthotopic nude mice liver cancer model with hepatic IR injury was established.

Nude mice liver cancer model with hepatic IR injury (IR injury group) was established by using MHCC97L-luciferase HCC cell line, the luciferin signal was examined by the Xenogen IVIS^®^ *in vivo* imaging system [25]. For the I/R injury group, the portal vein was clamped for 30 minutes to mimic the ischemia period during liver transplantation. After the 30 minutes ischemia, the tumor nodule with positive luciferin signal was implanted into the left lobe of liver. It has been demonstrated that the liver injury could not be obviously detected if the ischemia period was less than 30 minutes in our preliminary experiment. Generally speaking, 45 minutes could be applied for establishment of partial ischemia model [40]. However, the mice could not tolerate the surgical treatment if the ischemia period is too long in this study. Therefore, we selected 30 minutes as ischemia period. Cisplatin treatment started 2 weeks after the implantation. Tumor size was compared at 3 weeks and 4 weeks after cisplatin treatment by the Xenogen IVIS^®^ *in vivo* imaging system. After 4 weeks treatment, the mice were sacrificed and tumor samples were collected for further analysis.

### *In vitro* functional study

#### Cell culture

Human liver cancer cell lines (HepG2, Hep3B and Huh7) and human liver cell lines (MIHA and LO_2_) were purchased from the American Type Culture Collection (Manassas, VA, USA). The metastatic human liver cancer cell line MHCC97L was a gift which given from the Liver Cancer institute and Zhongshan Hospital of Fudan University, Shanghai, the People's Republic of China. All cells were grown in Dulbecco's modified eagle medium (DMEM) containing 10% FBS, 2 mM L-glutamine, 100 units/ml of penicilium and streptomycin (Life Technologies, Carlsbad, CA, USA).

#### Cell transfection and selection of stable transfectants

Full length of IP10 and PCDNA3.1(+) were transfected into PLC and MHCC97L, cell transfection was performed as previously study described [39]. The stable clone of MHCC97L-IP10 was selected using G418 at 0.4 mg/mL and the stable clone of PLC-IP10 was selected using G418 at 0.6 mg/mL. The selection period was 4 weeks and expression of target gene was confirmed using qRT-PCR and Western-blot.

#### 3-(4,5-Dimethyl thiazol-2-yl)-2,5-diphenyl tetrazolium bromide (MTT) assay

In order to explore the proliferation rate of HCC cells, MTT and colony formation assay were performed as previously described [39].

#### Colony formation assay

Colony formation assay was performed to compare the colony forming ability between different cell lines or with different types of drug administrations. Culture medium was changed every 3 days. After 2-week incubation, the colonies were washed with 1X PBS and fixed in 4% paraformaldehyde in 1X PBS. They were then stained with 0.5% crystal violet for 10 minutes at room temperature. The colonies were counted under a light microscope. The mean number of colonies was obtained from three independent experiments.

#### The function of rIP10 in HCC cell lines

Colony formation assay was performed to investigate whether the overexpression of IP10 in HCC cell lines could promote cell proliferation under the cisplatin environment. IP10 recombinant protein was applied to study the extracellular function of IP10 in HCC cell lines including Hep3B, Huh7, PLC and MHCC97L. Cells (5 × 10^3^cells/well) were seeded onto 6-well plates and incubated with different concentrations of cisplatin for 2 weeks. For the treatment group, 0.5 μg/ml IP10 recombinant protein was added into each well.

#### The intracellular function of IP10 in HCC cell lines

To investigate the intracellular function of IP10, IP10 overexpressed stable transfectants, PLC-IP10 and MHCC97L-IP10 were employed in colony formation assay.

#### The function of IP10 neutralizing antibody

To investigate the function of IP10 neutralizing antibody, MHCC97L-3.1 and PLC-3.1 with cisplatin administration alone were applied as the control groups. Meanwhile, MHCC97L-IP10 and PLC-IP10 with single cisplatin administration were also applied. MHCC97L-IP10 and PLC-IP10 with cisplatin and IP10 neutralizing antibody were considered as the combination treatment groups. The concentration of IP10 antibody for *in vitro* study is 0.5 μg/mL. The concentration of IP10 antibody was applied according to the manufacturer's instruction.

### Real time quantitative reverse transcription polymerize chain reaction (qRT-RCR)

The mRNA level of IP10 was determined by qRT-PCR as previously described [17]. The internal control was 18S ribosomal RNA (18S rRNA). The sequences of primers used were listed in Supplementary Table S1, S2 and S3.

### Enzyme-linked immunosorbent assay (ELISA)

The plasma samples were diluted 800 times and the medium for cell culture was without any dilution before detection. The concentration of IP10 was tested using ELISA kit according to instruction manual (AdipoGenInc, Incheon, Korea).

### Hematoxylin and eosin (H & E) staining

H&E staining was performed to determine the tumor necrosis in animal models. Method was described in our previous study [39].

### TUNEL assay

TUNEL staining was performed to determine the tumor cell apoptosis in animal models. Method was described in our previous study [39].

### Statistical analysis

The χ^2^ test was used to compare discrete variables. Independent-samples *T* test was used for comparison of continuous variables. *P* < 0.05 was considered to be statistically significant. Calculations were performed using SPSS computer software version 13 (SPSS, Chicago, IL, USA).

## SUPPLEMENTARY FIGURES AND TABLES


