# Supplementary material for: Interferon-gamma inducible protein 10 (IP10) induced cisplatin resistance of HCC after liver transplantation through ER stress signaling pathway
Source: Oncotarget. 2015 Aug 12;6(29):28042–56. doi: 10.18632/oncotarget.4832 (PMC4695043; doi:10.18632/oncotarget.4832)
Supplement: Supplementary file 1 [file oncotarget-06-28042-s001.pdf]

SUPPLEMENTARY FIGURES AND TABLES

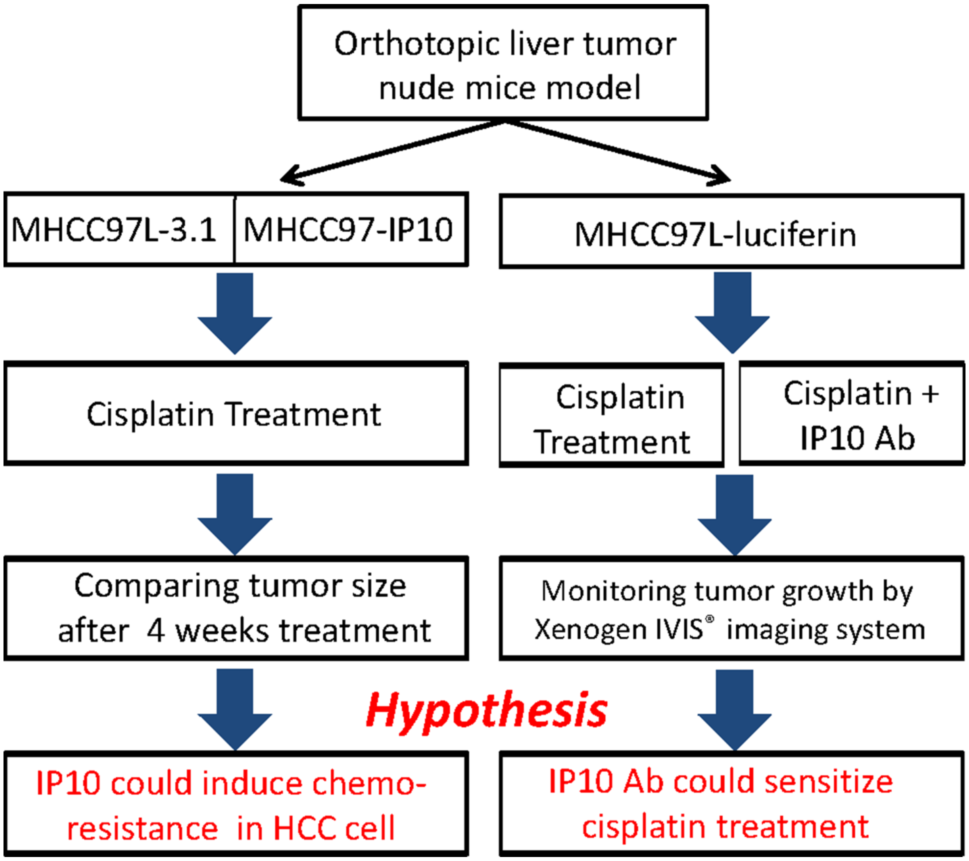

Supplementary Figure S1: Study designs for subcutaneous nude mice model.

(A)

(B)

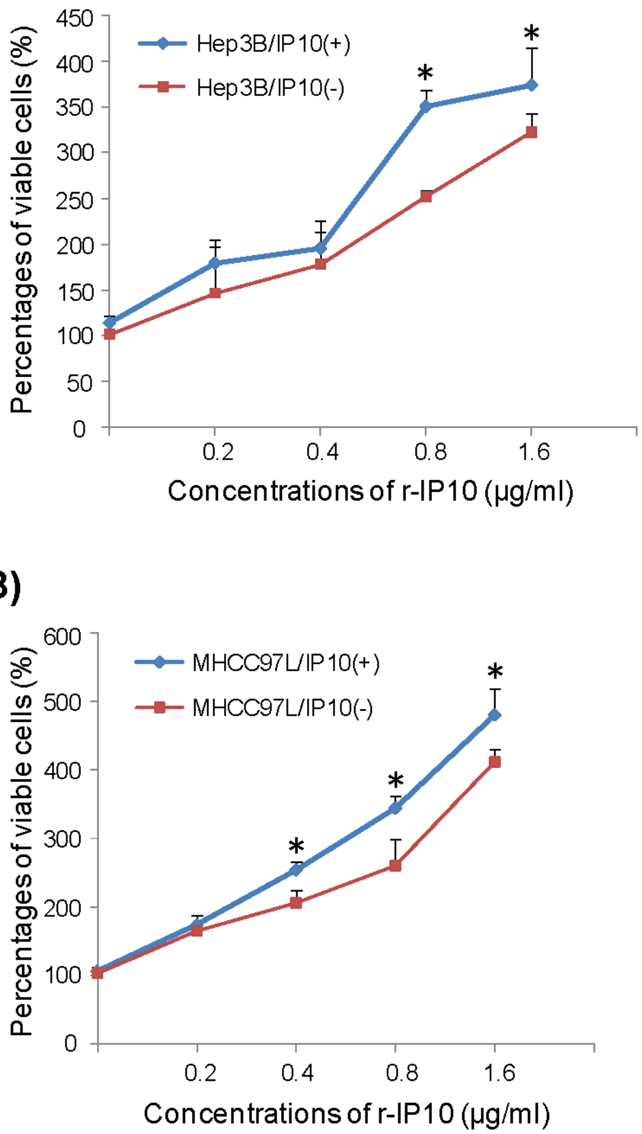

Supplementary Figure S2: The effect of IP10 recombinant protein on HCC cell lines *in vitro*. The effect of rIP10 administration on proliferation of Hep3B and MHCC97L for 72 hrs by MTT assay. *$P < 0.05$.

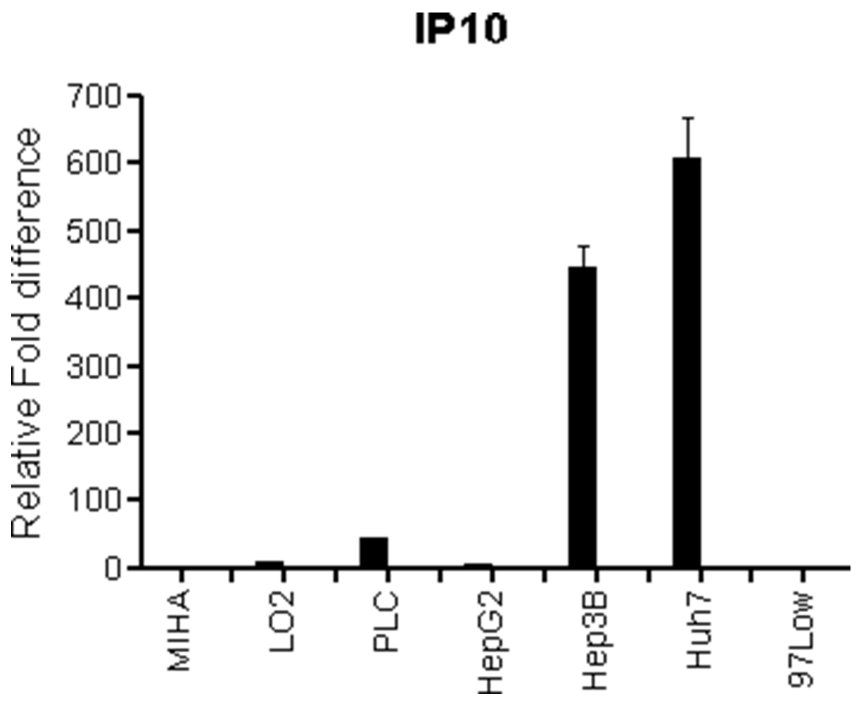

Supplementary Figure S3: The establishment of IP10 overexpressed stable transfectants. mRNA level of IP10 in PLC and MHCC97L stable transfectants by Q-PCR.

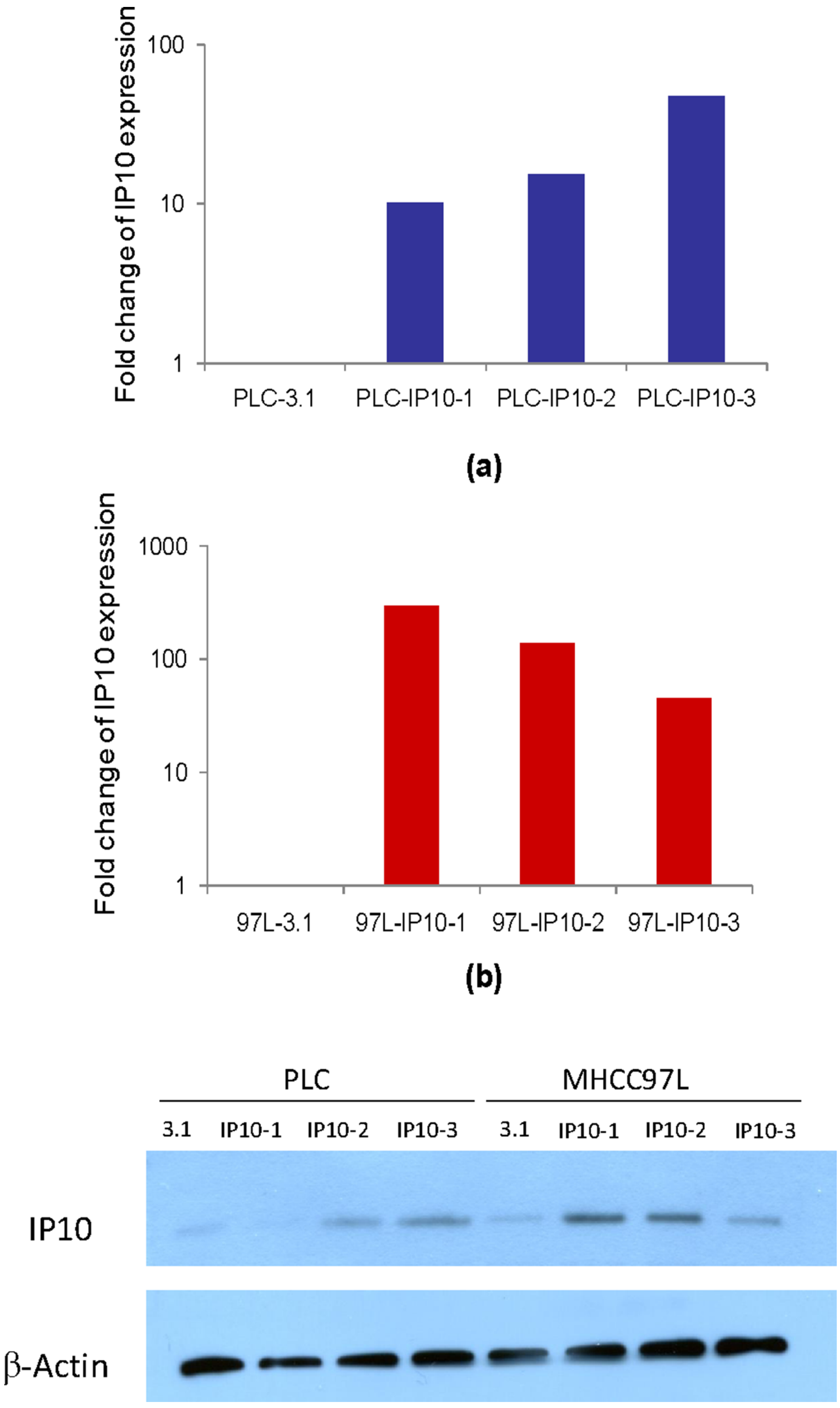

Supplementary Figure S4: Protein level of IP10 in PLC and MHCC97L stable transfectants by western blot.

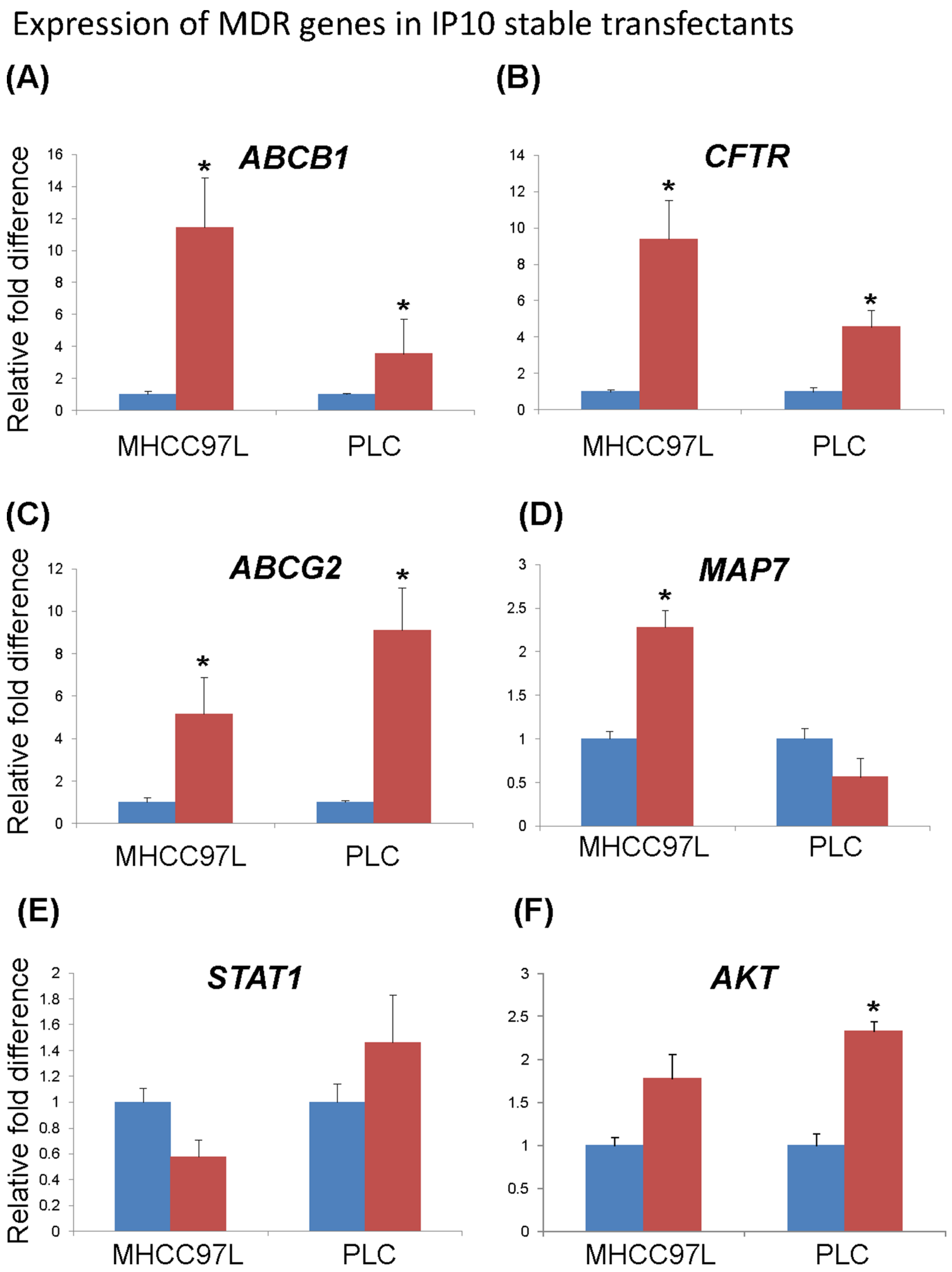

Supplementary Figure S5: Over expression of multidrug resistant genes in IP10 overexpressed stable transfectants. **A.** mRNA level of ABCB1 in IP10 stable transfectants and control HCC cell lines. $*P < 0.05$. **B.** mRNA level of CFTR in IP10 stable transfectants and control HCC cell lines. $*P < 0.05$. **C.** mRNA level of ABCG2 in IP10 stable transfectants and control HCC cell lines. $*P < 0.05$. **D.** mRNA level of MAP7 in IP10 stable transfectants and control HCC cell lines. $*P < 0.05$. **E.** mRNA level of STAT1 in IP10 stable transfectants and control HCC cell lines. $*P < 0.05$. **F.** mRNA level of AKT in IP10 stable transfectants and control HCC cell lines. $*P < 0.05$.

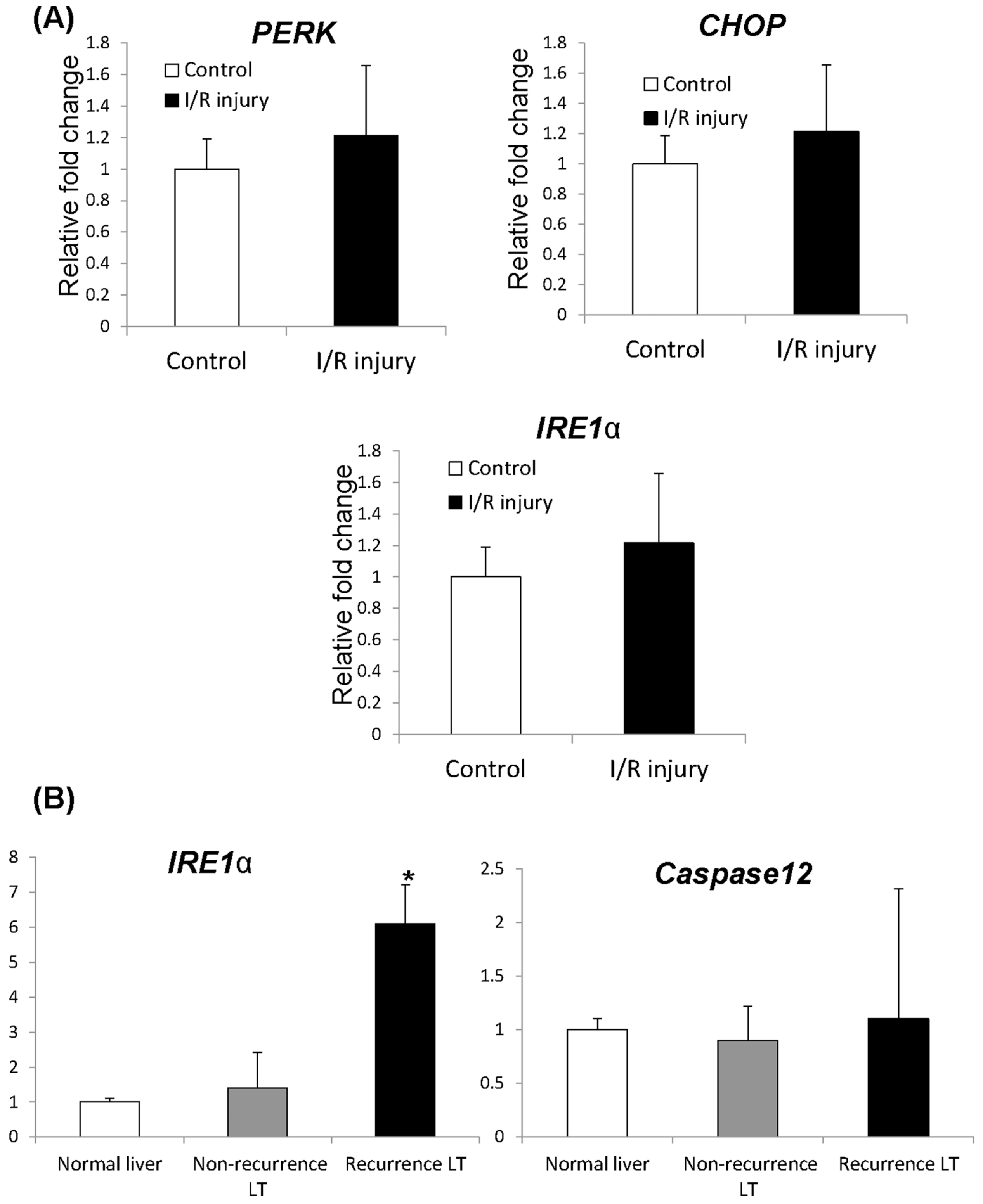

Supplementary Figure S6: Circulating mRNA expression levels of ER stress genes including Grp78, ATF4, ATF6, CHOP, IRE1alpha and Caspase12 in liver transplantation patients and its association with tumor recurrence.

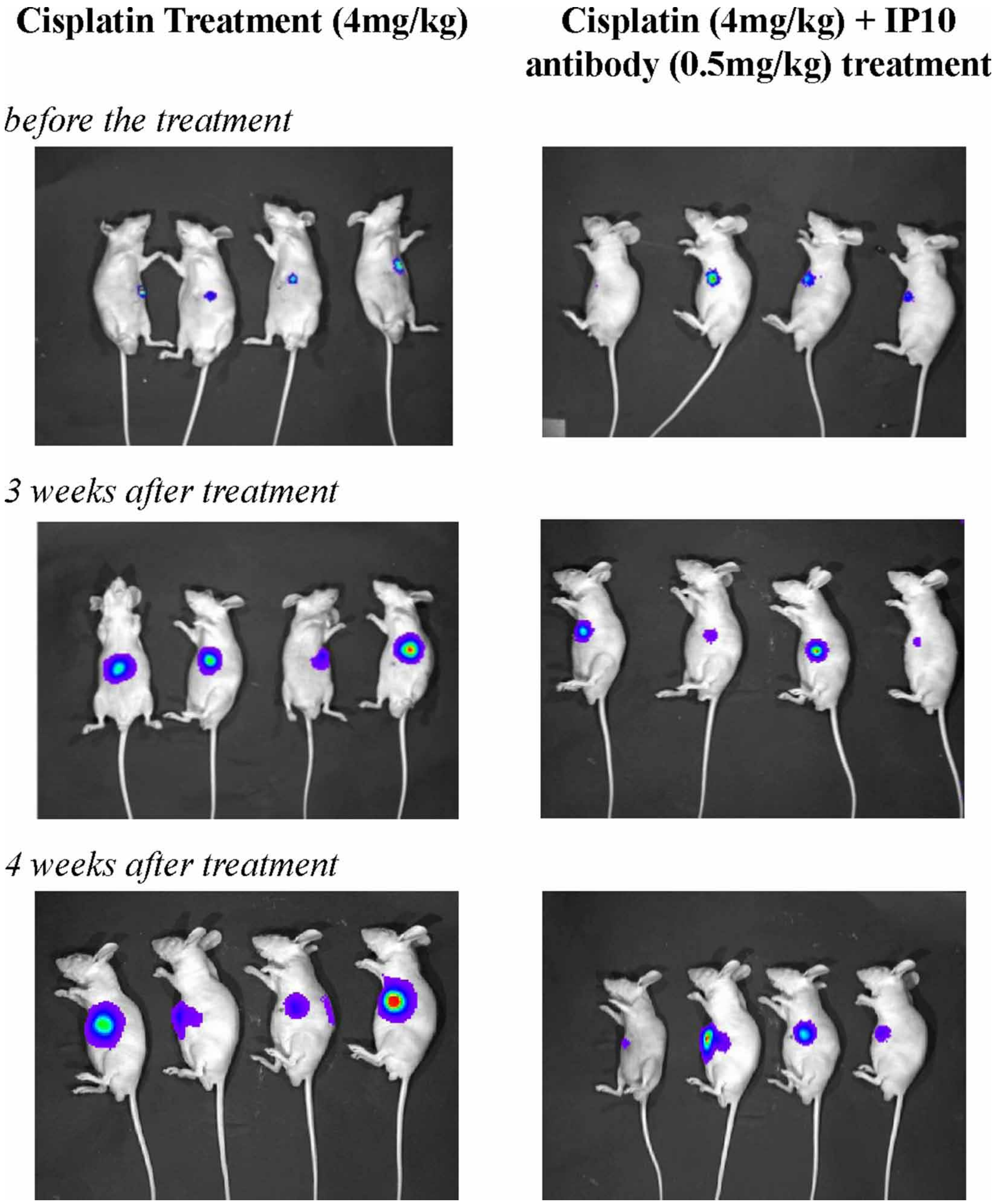

Supplementary Figure S7: Optical image of tumor nodules at the beginning of the treatment, 3 and 4 weeks after cisplatin/cisplatin+IP10 antibody treatments.

Supplementary Table S1: Primer design for testing rat samples

Gene name	Sequences
AKT	Forward: 5′ ACTCATTCCAGACCCACGAC 3′
	Reverse: 5′ CCGGTACACCACGTTCTTCT 3′
ABCB1	Forward: 5′ CGTTGCCTACATCCAGGTTT 3′
	Reverse: 5′ TGGAGACGTCATCTGTGAGC 3′
BCL-2	Forward: 5′ ATACCTGGGCCACAAGTGAG 3′
	Reverse: 5′ TGATTTGACCATTTGCCTGA 3′
ABCG2	Forward: 5′ AGTCCGGAAAACAGCTGAGA 3′
	Reverse: 5′ CCCATCACAACGTCATCTTG 3′
CFTR	Forward: 5′ CACCACTTGGAGCTGTCAGA 3′
	Reverse: 5′ CCTAGCAAGACAGGCTGGAC 3′
STAT1	Forward: 5′ AGTCACTCCCACCCACAAAG 3′
	Reverse: 5′ CTCTTGACCCAGTGGAGAGC 3′
Pyk2	Forward: 5′ GGCTTTCCTGCTGTTTCTTG 3′
	Reverse: 5′ CCCCACTGTAAGGCTCATGT 3′
IP10	Forward: 5′ AACGGTGGCTTTACCACTTG 3′
	Reverse: 5′ TCCCGATCTCCCTCTAGGAT 3′
LCN2	Forward: 5′ ATTTGCCCAGATGAAGATCG 3′
	Reverse: 5′ TCTCAGATGGTGGCTCTGTG 3′
AR	Forward: 5′ GCAAAACACCCCTAGCACAT 3′
	Reverse: 5′ GCCCATTTCAGGATTTCTCA 3′

Supplementary Table S2: Primer design for testing human samples or human HCC cell lines

Gene name	Sequences
AKT	Forward: 5′ CCTTGGCAAATGTGTGTTTG 3′
	Reverse: 5′ ATCAGCAGCAGCCTGAAAAT 3′
ABCB1	Forward: 5′ TGCTGATGGAGAAGTTGTGC 3′
	Reverse: 5′ GTCAGCCTGTCCTTTGAAGC 3′
MAP7	Forward: 5′ CCAAGCTTTTCCAGAGCAAC 3′
	Reverse: 5′ CTCTGGGATCAACACCACCT 3′
ABCG2	Forward: 5′ TCAAAATCCCATCTGCTTCC 3′
	Reverse: 5′ CTGGGACCTTCATTTCTCCA 3′
CFTR	Forward: 5′ CGAGTAGCTGGGTCTTCAGG 3′
	Reverse: 5′ TTTAGGCTGACAGGGTGAGG 3′
STAT1	Forward: 5′ CCCAAGAAGTGGGAGAATCA 3′
	Reverse: 5′ CAACTCAGAGGCCTTTCCTG 3′
IP10	Forward: 5′ TTTCCCCAAGTCTTTCATGG 3′
	Reverse: 5′ CCTCTGTGTGGTCCATCCTT 3′
GRP78	Forward: 5′ AGGTGTGAGCCACTGTACCC 3′
	Reverse: 5′ TAGATCCCCGCATTTCAGTC 3′
ATF6	Forward: 5′ GAGCAACTGGGAAAATCCAA 3′
	Reverse: 5′ TCAAGATGCATGTGCAGTGA 3′
PERK	Forward: 5′ CCAGCCTTAGCAAACCAGAG 3′
	Reverse: 5′ TGCCCTAAAGGGACACAAAC 3′
CHOP	Forward: 5′ AGTGCCACGGAGAAAGCTAA 3′
	Reverse: 5′ CCATACAGCAGCCTGAGTGA 3′
IRE1α	Forward: 5′ TGTGGGGTCCTTTAGTTTGC 3′
	Reverse: 5′ AAGAACTGCAGCCTCCAAAA 3′
Caspase12	Forward: 5′ ACTGTGGGGAAGGAGTTGTG 3′
	Reverse: 5′ AAAAACAAAACCCTGCAACG 3′

Supplementary Table S3: Primer design for testing mice samples

Gene name	Sequences
IP10	Forward: 5′ GGATGGCTGTCCTAGCTCTG 3′
	Reverse: 5′ ATAACCCCTTGGGAAGATGG 3′
GRP78	Forward: 5′ AGTGGTGGCCACTAATGGAG 3′
	Reverse: 5′ CAATCCTTGCTTGATGCTGA 3′
ATF6	Forward: 5′ CCTGCAGAGGTCAGAAAAGG 3′
	Reverse: 5′ AGCCAGGGCTACACAGAGAA 3′
PERK	Forward: 5′ ACTGTAGGGCGGTTCAAATG 3′
	Reverse: 5′ ATTTCCAACCCCCATTCTTC 3′
CHOP	Forward: 5′ GGGATCTCTCCAGGGTCTTC 3′
	Reverse: 5′ CCCTTGGTCTTCCAGTGTGT 3′
IRE1α	Forward: 5′ TTTCCCATGGATGGAATTGT 3′
	Reverse: 5′ CATCCATTCAGAAAGCAGCA 3′
Caspase12	Forward: 5′ GCAGGCATAGCATGTCTTCA 3′
	Reverse: 5′ TCCTTGTGCACACTCAGAGG 3′
